# Comparing multi- and single-exposure speckle contrast optical spectroscopy methods as estimators of blood flow in the diffuse regime

**DOI:** 10.1117/1.JBO.31.2.025002

**Published:** 2026-02-09

**Authors:** Sean Aleman, Arnold D. Estrada, Ashwin B. Parthasarathy

**Affiliations:** a University of South Florida, Department of Medical Engineering, Tampa, Florida, United States; b SPKL, LLC, Tampa, Florida, United States; c University of South Florida, Department of Electrical Engineering, Tampa, Florida, United States

**Keywords:** speckle, diffuse speckle, diffuse correlation spectroscopy, speckle contrast optical spectroscopy, speckle plethysmography, blood flow

## Abstract

**Significance:**

Speckle contrast optical spectroscopy (SCOS) and speckle plethysmography (SPG) are increasingly used to measure deep tissue blood flow from tissues. However these methods derive flow from measurements at a single exposure duration, which could introduce errors due to inefficient choice of a single integration time, changes in speckle averaging factors (β), and noise.

**Aim:**

The aims are to compare and evaluate the robustness of single- and multi-exposure speckle contrast methods in estimating blood flow changes under experimental conditions such as β mismatch, noise, and pulsatile flow.

**Approach:**

Speckle visibility was simulated at 10,000 logarithmically spaced exposure durations (0.01 to 10 ms), incorporating ±5 to 15% noise, using a semi-infinite diffusion model at different physiologically relevant steady state and pulsatile flow rates. Speckle visibility was used to estimate blood flow changes using both the multi-exposure approach (non-linear fitting of the full curve) and conventional single exposure approach at 0.1, 1, and 5 ms durations (SCOS, SPG, and look-up-table), under different noise conditions and mismatches in β.

**Results:**

Multi-exposure speckle imaging (MESI) maintained <1% mean error despite β mismatch or noise while maintaining ≤12% pulsatile-flow error at ±5% noise. Single-exposure methods showed errors up to 90% for β mismatch/noise and ≥22% pulsatile-flow errors.

**Conclusions:**

MESI outperformed single-exposure approaches in estimating blood flow changes by reducing β bias, minimizing noise sensitivity, and accurately tracking pulsatile dynamics.

## 1 Introduction

Diffusion of coherent near-infrared light through tissue enables quantitative, non-invasive measurement of tissue perfusion or blood flow from structures a few centimeters below the surface. Coherent light undergoes multiple scattering events as it propagates through tissue, producing a speckle pattern that can be imaged and analyzed to compute indices of blood perfusion in tissue.^1^ The most common implementation of this approach is diffuse correlation spectroscopy (DCS), which measures the temporal intensity fluctuations of the diffuse speckle pattern to quantify blood flow *in vivo* as a blood flow index (F).^2^^–^^4^ Blood flow indices measured with DCS have been validated in various tissue perfusion studies^5^^,^^6^ and the technique has been applied to measure perfusion in organs/tissues such as the brain,^3^^,^^4^^,^^7^ muscle,^8^ breast,^9^^,^^10^ and spinal cord.^11^^,^^12^ DCS instruments typically consist of a long coherence length laser source, fiber-optic cables to deliver/collect light to/from tissue, and single photon-counting avalanche photodetectors to record intensity of reflected diffuse speckle.^3^^,^^6^^,^^13^ Although single photon-counting avalanche photodetectors provide sufficient sensitivity to detect low light levels (a few pW) emanating from a single speckle, they are expensive, bulky, and require fast time-tagging electronics.

More recently, we and others have demonstrated that diffuse blood flow measurements can be realized with simpler detection systems such as photodiodes^14^^,^^15^ and cameras.^16^^–^^19^ Broadly termed speckle contrast optical spectroscopy (SCOS), speckle plethysmography (SPG), or integrated diffuse speckle contrast spectroscopy, these methods estimate flow from spatial and temporal speckle pattern fluctuations. Early SCOS approaches demonstrated the feasibility of capturing full speckle “visibility” curves across multiple camera exposure durations to characterize blood flow dynamics in detail.^19^ Newer implementations of SCOS acquire and calculate the speckle contrast (variance) at a single fixed exposure time, in part to reduce instrumentation complexity.

Here, we focus on conventional SCOS and SPG implementations that acquire and compute blood flow index from images acquired at a single camera exposure duration. SCOS captures both pulsatile blood volume changes [photoplethysmography (PPG)] and microvascular blood flow index (BFI) simultaneously^19^^,^^20^ and is a diffuse optical variant of speckle contrast imaging designed to probe deeper tissues by combining speckle contrast analysis with photon diffusion modeling.^19^ Typically, spatial variance measured with SCOS are biased by noise (e.g., shot noise, read noise, and quantization noise),^21^ which can be estimated and corrected to improve the fidelity of the estimated blood flow index.^19^ Nevertheless, when implemented as a single-exposure time measurement, SCOS is limited to quantitative estimates of relative blood flow changes. Similarly, SPG adapts speckle contrast imaging for pulsatile blood flow monitoring (analogous to photoplethysmography). By tracking the speckle contrast fluctuations synchronized to the cardiac cycle, SPG provides a measure of the arterial pulse and has been shown to have higher signal-to-noise ratio compared with traditional PPG.^22^^,^^23^ Like SCOS, SPG analyses typically use a fixed camera integration time and yield relative pulse waveforms rather than an absolute flow index.

Despite the relative instrumentation simplicity, blood flow measurements with single-exposure speckle contrast measurements have some notable limitations. First, they inherently provide only a snapshot of the speckle decorrelation process. It is difficult to estimate baseline indices of blood flow without capturing the full speckle visibility curve. More significantly, the dynamic range of blood flow changes measured with single-exposure methods are limited and depend on the baseline flow levels. In contrast, by sampling the speckle pattern at several integration times, multi-exposure methods^14^^,^^24^ can record the full speckle visibility curve and thereby improve flow estimation. Second, the fidelity of blood flow estimates depend in part on how well the single-exposure techniques can characterize the speckle averaging factor β. Multi-exposure methods can fit for β from the full speckle visibility curve. Notably, these limitations of single exposure speckle measurements vis-à-vis multi-exposure have been studied before within the context of speckle-based wide-field blood flow imaging instruments.^25^^,^^26^ However, these differences have not yet been systematically evaluated for diffuse (deep tissue) blood flow measurements. In this paper, we address this gap by performing a comprehensive simulation-based comparison of multi-exposure speckle imaging (MESI) with SCOS, SPG, and a single-exposure look-up table (LUT) method, a pre-computed calibration table relating speckle contrast, β, and exposure duration to BFI, across a range of β values and noise levels. We evaluate each method’s accuracy in quantifying relative blood flow changes and its robustness to β misestimation and measurement noise.

## 2 Methods

### 2.1 Simulation and Data Generation—Forward Model

We developed a MATLAB-based simulation to compute speckle variance $K^{2}$ as a function of camera exposure time and to evaluate methods for estimating the BFI. Speckle visibility curves were simulated using the semi-infinite diffuse speckle model with physiologically realistic parameters (wavelength λ=785  nm, absorption coefficient $\mu_{a}=0.1\;{\mathrm{cm}}^{-1}$, reduced scattering coefficient $\mu_{s}^{\prime }=10\;{\mathrm{cm}}^{-1}$, refractive index of tissue $n_{\text{tissue}}=1.4$, refractive index of the medium $n_{\text{medium}}=1$, and source-detector separation ρ=3  cm). BFI values ranged from $0.5\times 10^{-8}∼2\times 10^{-8}\;{\mathrm{cm}}^{2}/s$, representative of typical blood flow rates in adults. This range serves to test the sensitivity of speckle contrast to changes in BFI. The normalized speckle variance $K^{2}(T)$ at exposure time T was modeled as

$$K^{2}(T,F)=\beta \frac{2}{T}\int_{0}^{T}(1-\frac{\tau }{T})|g_{1}(\tau ,F){|}^{2}d\tau ,$$ (1)

where β is the speckle averaging factor, F is the blood flow index, and $g_{1}(\tau ,F)$ is the normalized electric field temporal autocorrelation function. We modeled the normalized electric field autocorrelation function using the semi-infinite geometry solution to the correlation diffusion equation from DCS literature.^2^^,^^3^ The speckle variance expression can be evaluated in closed form under the diffusion approximation, enabling direct computation of $K^{2}$.^14^^,^^24^ We leveraged this analytical solution in our forward model to efficiently generate expected $K^{2}(T)$ curves for given BFI values and optical parameters.

Speckle visibility curves were generated over 10,000 logarithmically spaced exposure times from 10  μs (short exposure) to 10 ms (long exposure). From this span, we selected three representative exposure durations—0.1, 1, and 5 ms—to serve as the single-exposure data in subsequent analysis. These three exposures sample short-, intermediate-, and long-integration regimes to capture flows of varying speeds and serve as the inputs to the various flow estimation methods for comparison. Higher BFI values (faster blood flow) lead to a quicker decay of $K^{2}$ with exposure time, whereas lower BFI (slower flow) yields a more gradual decay. Data were also simulated for different values of β, ranging from 0.1 to 0.5.

### 2.2 Noise Handling

To assess how single- and multi-exposure methods handle intrinsic fluctuations in speckle contrast, we introduced multiplicative Gaussian noise into the simulated data. This noise was not intended to mimic biases such as shot noise or readout noise but rather to emulate physiological and statistical variability inherent in speckle measurements. Such variability could arise from limited ensemble averaging, dynamic scattering, and tissue motion and may persist even after standard noise correction procedures (e.g., SCOS-style noise subtraction). Specifically, we introduced zero-mean Gaussian noise into the simulated $K^{2}$ data at three levels: ±5%, ±10%, and ±15%, using the expression

$$K_{\text{noisy}}^{2}=K_{\text{true}}^{2}\times (1+\alpha N(0,1)),$$ (2)

where N(0,1) is a standard normal variable, and α is the noise level (0.05, 0.10, or 0.15).

We conducted 10,000 random samplings of this distribution per BFI and noise level. For each trial, a noisy $K^{2}(T)$ curve was generated and sampled at 0.1, 1, and 5 ms. These values were processed by each flow estimation method (described below), and the resulting percent changes were used to compute mean error and standard deviation.

### 2.3 BFI Estimation Methods

Four methods were implemented to estimate BFI from the speckle data: MESI, LUT, SCOS, and SPG. Each method leverages a different numerical approach and set of assumptions to solve for the BFI from speckle contrast measurements.

Multi-exposure speckle imaging: The MESI approach uses non-linear least-squares fitting across all available exposure durations to simultaneously estimate BFI and β using the analytical model described earlier.^14^ Briefly, the multi-exposure approach fits a speckle visibility curve $\left[K^{2}(T)\right]$ to an analytical model that is appropriate to the measurement geometry. Prior implementations of the MESI approach for the diffuse geometry include Biswas et al.^14^ and Valdes et al.^19^ In this paper, the simulated speckle visibility curve (with addition of noise) was fit to the analytical expression described in Eq. (1), the closed form solution of which has been described elsewhere.^14^^,^^24^^,^^27^ The tissue absorption coefficient and reduced scattering coefficient were assumed to be $0.1\;{\mathrm{cm}}^{-1}$ and $10\;{\mathrm{cm}}^{-1}$, respectively. The refractive index of tissue and medium were 1.4 and 1, respectively, and source-detector separation was 3 cm. Fits were implemented in MATLAB using unconstrained non-linear optimization (fminsearch) to estimate β and blood flow index. In our implementation, the initial guess for BFI was set to a mid-range value ($1\times 10^{-8}\;{\mathrm{cm}}^{2}/s$) and for the guess for β was 0.5.

LUT approach: The LUT method uses a precomputed calibration table to directly map measured speckle contrast to BFI under different exposure times and β values. This was used to invert single exposure data to the BFI. We generated a dense LUT by numerically solving the theoretical speckle variance $\left[K^{2}(T)\right]$ at the three single exposure times, spanning a BFI range from $0.1\;\times 10^{-8}$ to $8\times 10^{-8}\;{\mathrm{cm}}^{2}/s$. The LUT comprised 790 flow values (BFI grid points) and 51 β values (from 0 to 0.5, in 0.01 increments), ensuring fine interpolation and robust inversion across all plausible measurement conditions. For a given measurement, the algorithm interpolates within this table to find the BFI that best matches the observed $K^{2}$. This parameter space was uniformly sampled (Δβ=0.01, $\Delta \mathrm{BFI}\approx 1\times 10^{-10}\;{\mathrm{cm}}^{2}/s$), with no sparsely populated regions, providing continuous coverage across the LUT domain. Each $K^{2}-\mathrm{BFI}$ curve was verified to be monotonic for all β, ensuring unique and stable inversion for every input measurement. This approach is fast (no iterative fitting) and was implemented via MATLAB.

Speckle contrast optical spectroscopy: SCOS estimates the blood flow index (BFI) by analyzing the speckle contrast of a single-exposure image, leveraging the inverse relationship between speckle contrast and decorrelation time. The BFI in SCOS is computed using the relation^20^

$$\mathrm{BFI}=\frac{1}{K^{2}}.$$ (3)

We apply this estimator at each of the three exposure times independently to compare the effect of exposure duration on the results.

Speckle plethysmography: Similarly, the SPG method uses a single-exposure time-series analysis to infer flow. SPG typically calculates the blood flow index as^23^

$$\mathrm{BFI}=\frac{1}{2TK^{2}}.$$ (4)

Similar to SCOS, we apply this estimator at each of the three exposure times independently to compare the effect of exposure duration on the results.

After applying these methods, we computed the percentage change in flow relative to the baseline condition (${\mathrm{BFI}}_{\text{baseline}}=1.0\times 10^{-8}\;{\mathrm{cm}}^{2}/s$). For each estimation method and each true BFI, percentage change in flow was calculated as $\mathrm{rBFI}=\frac{{\mathrm{BFI}}_{\mathrm{est}}-{\mathrm{BFI}}_{\text{basline}}}{{\mathrm{BFI}}_{\text{baseline}}}\times 100$. This yields flow changes of 0% at baseline, −50% for the lowest BFI (half the baseline), and +50% and +100% for the higher BFIs (1.5× and 2× baseline, respectively). Finally, to quantify the absolute accuracy, we evaluated the % error in BFI estimates as ${\mathrm{BFI}}_{\text{error}}=\frac{\left|{\mathrm{BFI}}_{\mathrm{est}}-{\mathrm{BFI}}_{\text{true}}\right|}{{\mathrm{BFI}}_{\text{true}}}\times 100\%$. For example, if true $\mathrm{BFI}=0.5\times 10^{-8}$ and the method estimated $0.6\times 10^{-8}$, the error would be 20%. We computed these errors for each flow level, each assumed β, and each noise level.

To summarize performance, we computed the averages of these errors in two ways: (1) across the non-baseline flow levels (to get an overall percentage error for each method under given conditions) and (2) across the three exposure times (to see if certain exposure durations contribute more to error). These error metrics allow comparison of methods in terms of accuracy and robustness. We compare the three methods in terms of their ability to track known flow changes. The percentage change outcomes indicate how well each method preserves linearity of the flow-response relationship.

### 2.4 Simulating Effects of β Mismatch

To directly assess sensitivity to the speckle averaging factor, we analyzed the flow changes measured with MESI and the LUT-based method. Briefly, we inverted the speckle visibility data simulated with β<0.5, using MESI and the LUT method. In MESI, β was treated as a free parameter and estimated simultaneously with BFI in the non-linear least-squares fitting process. For the LUT method, β must be assumed; here, we fixed β=0.5 for inversion while varying $\beta_{\text{true}}$ in the forward simulation. This simulates a scenario where the beta is expected to be 0.5 (as in the initial guess or the LUT column), but the synthetic visibility data were generated to represent an experimental condition where $\beta_{\text{true}}\neq 0.5$. In practice, this corresponds to what would be observed if experimental data were collected under slightly altered coupling or coherence conditions. Such a scenario may occur due to the changes in the coupling between the probe and the skin (e.g., due to motion), changes in laser coherence length or stability, motion in the fiber optic cable, etc. This mismatch is important to characterize to evaluate how β mismatches can impact measured blood flow changes. The condition of no β mismatch ($\beta_{\text{true}}=0.5$) corresponds to using the measured β in the inversion

### 2.5 Pulsatile Flow Simulation

In addition to the four discrete BFI values, we also characterized the effects of flow estimation with the different methods for typical pulsatile flow. Here, we simulated speckle visibility curve data for a 10-s duration and 10-Hz temporal resolution of pulsatile blood flow indices derived from an *in vivo* DCS measurement from a ior study.^13^ For each flow index, the forward model [Eq. (1)] was applied to compute the speckle visibility curve at the 10,000 exposure durations, and noise was added. These simulated speckle visibility curves were fit to the diffuse MESI model to estimate the pulsatile flow indices. This derived a frame-by-frame estimation of both BFI and β values. Similarly, single-exposure estimates of flow changes were computed using the LUT, SCOS, or SPG methods at 0.1, 1, and 5 ms exposure durations. Multiplicative noise of ±1% and ±5% was applied, and 5000 trials were run per methods described above. Performance was evaluated by calculating the mean and maximum absolute error in tracking the relative blood flow waveform.

## 3 Results

**Fig. 1:**
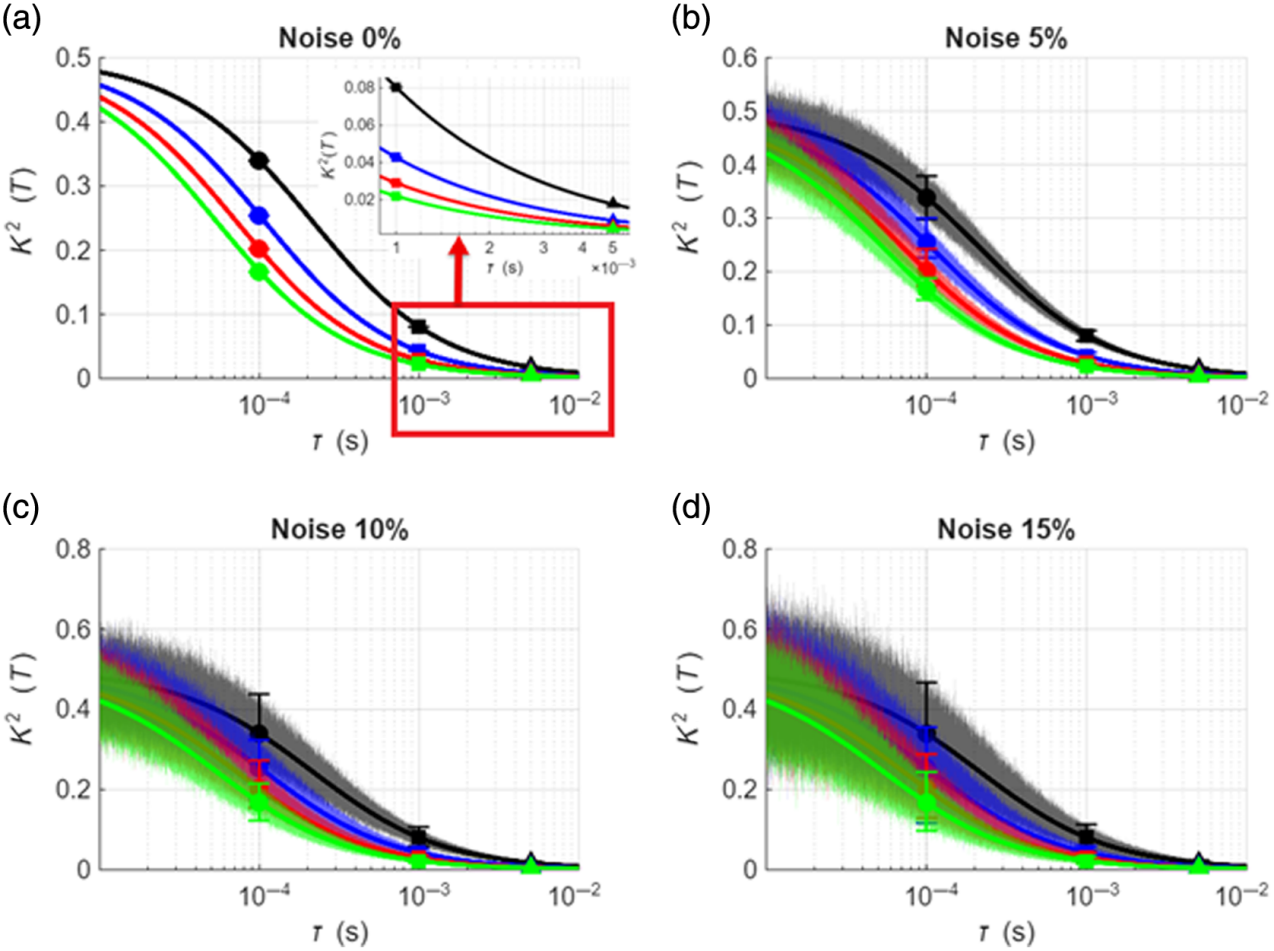
(a) Simulated speckle visibility curves [$K^{2}$ (T)] for four BFI values ($0.5\times 10^{-8}$, $1.0\times 10^{-8}$, $1.5\times 10^{-8}$, and $2.0\times 10^{-8}\;{\mathrm{cm}}^{2}/s$) and β=0.5. Circle, square, and triangle markers denote in turn 0.1, 1, and 5 ms exposure durations, respectively. (b)–(d) Visibility curves at 5%, 10%, and 15% noise levels.

We first describe the data used to perform a comparative analysis between MESI and single exposure methods for estimating blood flow. Figure 1(a) shows simulated speckle visibility curves for different flow rates with zero noise. The black, blue, red, and green lines denote flow rates of $0.5\times 10^{-8}$, $1\times 10^{-8}$, $1.5\times 10^{-8}$ and $2\times 10^{-8}\;{\mathrm{cm}}^{2}/s$, respectively. All curves were generated for β=0.5. The markers circle, square, and triangle correspondingly denote speckle variance at exposure times of 0.1, 1, and 5 ms, respectively. Figures 1(b)–1(d) show the corresponding visibility curves at 5%, 10%, and 15% noise, respectively. The effect of sampling the full speckle visibility curve at a few exposure times is evident on the inlay image in Fig. 1(a). For example, at 0.1-ms exposure, the variance values for different BFIs are still relatively close together (only about a twofold difference between the lowest and highest BFI). However, at 1 ms, they diverge significantly (approximately fourfold difference between the lowest and highest BFI). Thus, with single-exposure models, the same blood flow change (a “true” 4× change in blood flow from $0.5\times 10^{-8}$ to $2\times 10^{-8}\;{\mathrm{cm}}^{2}/s$) would be estimated as a 2× blood flow increase at 0.1 ms and a 4× increase at 1 ms. This illustrates the non-linear relationship between the exposure times and contrast. These differences are compounded significantly in the presence of noise.

### 3.1 Effects of Noise and β Mismatch on Estimation of Blood Flow Index

We first compared the accuracy of single versus multi-exposure methods to estimate the blood flow index under different noise and β mismatch conditions. Only the LUT method was used for single-exposure comparisons because SCOS and SPG yield flow indices in arbitrary units and are better suited for quantitative measurements of relative blood flow. Thus, flow indices measured with SCOS/SPG cannot be directly compared with MESI. On the other hand, the LUT approach provides BFI in identical physical units, enabling a one-to-one comparison with MESI for evaluation of noise and β effects.

**Fig. 2:**
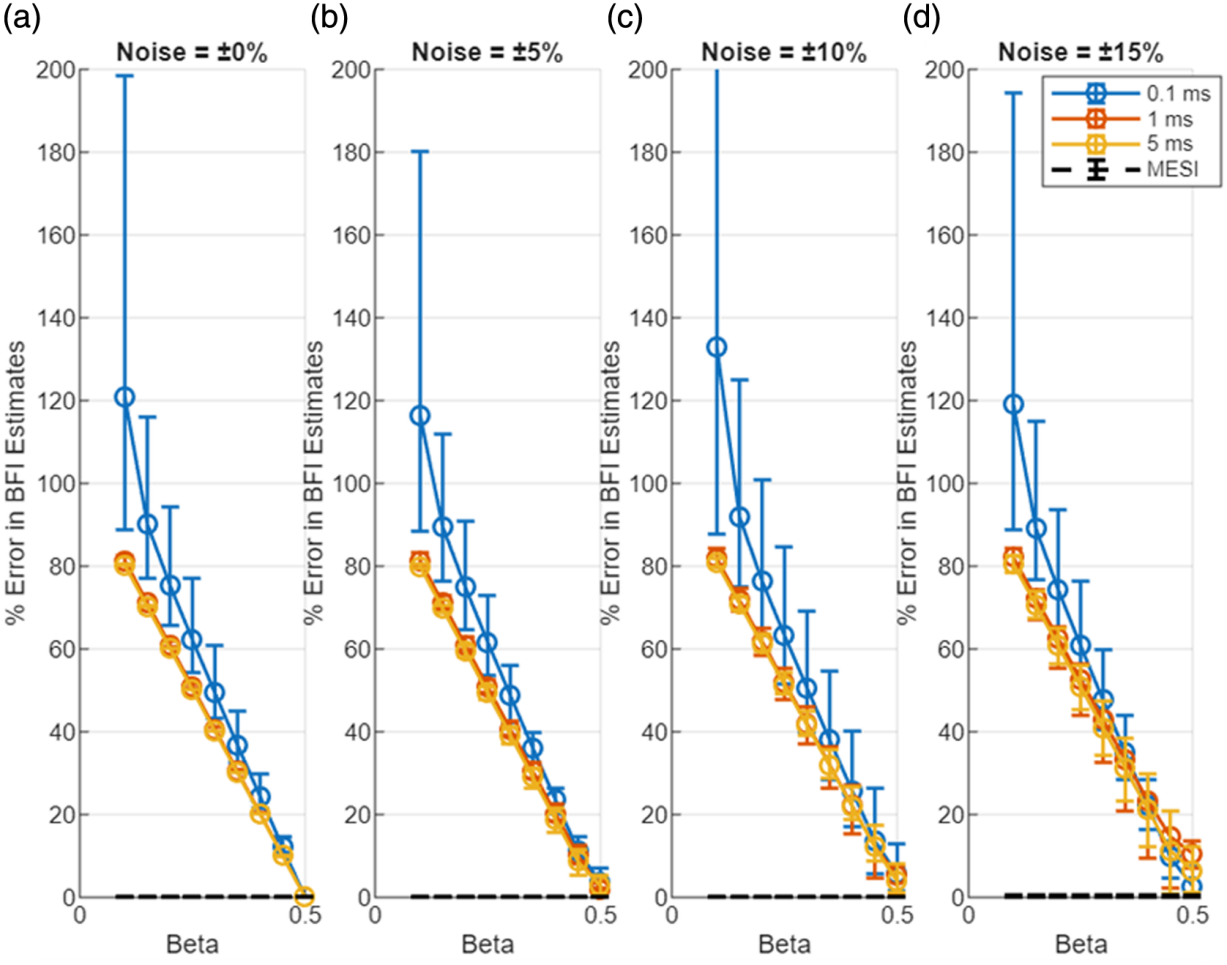
Effect of mismatch in β between measurement and analysis in estimates of blood flow index with MESI and the single-exposure LUT methods. The X-axis indicates the β used to generate the visibiliy curves (β=0.5 was used in the analysis). The Y-axis is the % error in BFI estimate. (a)–(d) Errors for different levels of noise.

**Fig. 3:**
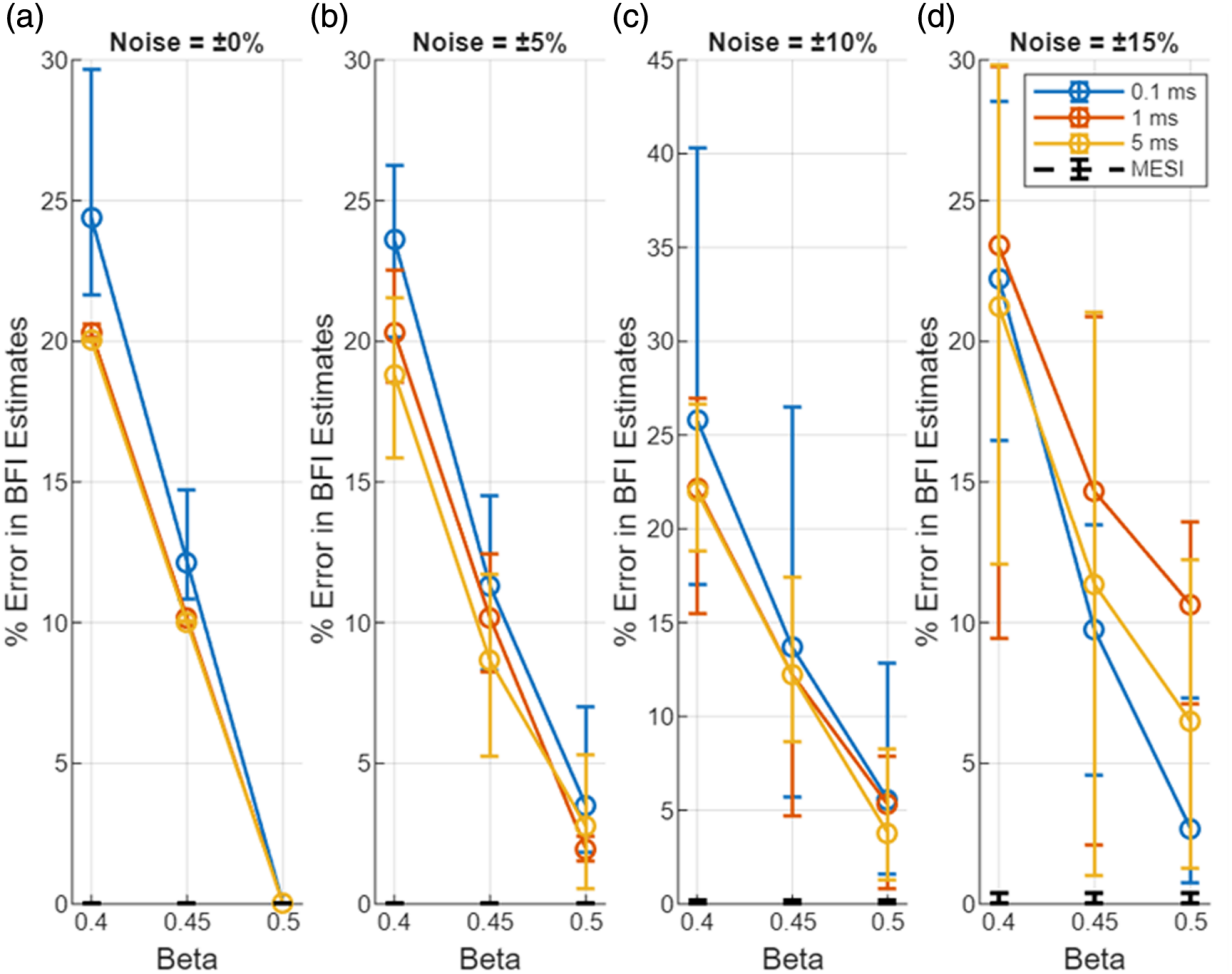
Zoomed-in view of BFI estimation error due to mismatch in β between measurement and analysis in estimates of blood flow index with MESI and the single-exposure LUT methods. The X-axis indicates the β used to generate the visibiliy curves (β=0.5 was used in the analysis). The Y-axis is the % error in BFI estimate. (a)–(d) Errors for different levels of noise. β=0.4 to 0.5 reflect the realistic β changes during an experiment.

Unless otherwise stated, all error bars in the figures represent the full range of values (minimum–maximum) across 10,000 simulation trials, and markers denote the mean of those trials. Figure 2 shows the error in the blood flow index estimated with the LUT method under increasing noise. Here, the % error in BFI (${\mathrm{BFI}}_{\text{error}}$) is plotted as a function of β mismatch for the three representative exposure durations (0.1, 1, and 5 ms); each subplot corresponds to a different noise level. β on the X-axis refers to the value used to generate the data with the forward model, and β=0.5 was used in the LUT inversion. Each data point represents the mean error over 10,000 trials, with error bars indicating the range of estimated errors. BFI errors for the different “true” flow values were averaged. The markers represent the average error across all trials, and the error bars represent the range of estimated errors. Figure 3 presents a focused view of these error trends for a smaller range of β (0.4 to 0.5, which is arguably experimentally realistic).

**Fig. 4:**
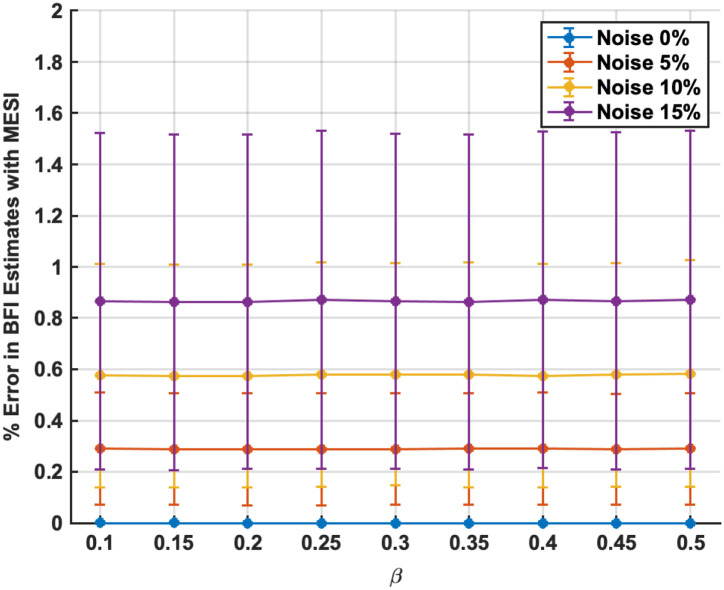
MESI robustness to estimating BFI accurately with both β mismatch (X-axis) noise levels (0%, 5%, 10%, and 15% in blue, red, yellow, and magenta curves, respectively).

Together, Figs. 2 and 3 illustrate the degradation in BFI accuracy due to noise for single-exposure LUT method. When there is no β mismatch and no noise, the LUT method accurately estimates the bood flow index. However, the introduction of noise in the measurement increase the bias and error in BFI estimates. Even when the assumed β matches the true value (β=0.5), the LUT results in Figs. 2(b)–2(d) show that mean BFI error systematically increases with rising multiplicative noise—from ∼4% at ±5% noise to ≈11% at ±15%. These trends worsen when β mismatch is introduced. At moderate noise levels (up to ±10%), both the 1- and 5-ms single-exposure estimators already exhibit significant errors (>15% mean error) even for small β mismatches in the 0.4 to 0.5 range. The shortest exposure (0.1 ms) is even more susceptible to noise (error curves rising more steeply, though not fully shown in the focused; Fig. 3). These findings underscore the inherent limitations of single-exposure approaches: simply optimizing the exposure duration or calibrating β cannot fully counteract the impact of measurement noise on accuracy. These trends, also reflected in the widening error bars of Figs. 2 and 3, arise because multiplicative noise perturbs $K^{2}$ non-linearly and introduces bias in the inversion process. In contrast, MESI maintains <2% mean error across the same noise conditions (Fig. 4), underscoring its noise resilience even when β is perfectly calibrated. As noise increases (toward ±15%), the errors for single-exposure methods continue to grow, and the trial-to-trial variability becomes very large—in fact, in some trials, the LUT method yielded wildly erroneous BFI values when noise pushed the speckle contrast outside the calibrated range. With ±15% added noise and a substantial β mismatch, a single 5-ms exposure could occasionally produce BFI estimates more than double the true value (errors exceeding 100%).

Figure 4 plots the mean percentage error in the recovered BFI using MESI when multiplicative noise of 0%, 5%, 10%, and 15% is introduced, for different instances of β mismatches. The accuracy of BFI estimates with MESI are not dependent on the β mismatch even for the highest noise level. This is primarily due to the robustness of the non-linear fitting, which allows the MESI approach to converge to the true β value. The % error in BFI estimates increases marginally with noise; the average % error increases from close to zero under no noise to ∼0.9% for 15% noise. Furthermore, MESI produced no extreme outlier estimates in any of the 10,000-trial simulations—we did not encounter a single instance where MESI’s BFI estimate erred by more than ∼1.6% in an individual trial, even at ±15% noise. MESI’s use of multiple exposure times in a simultaneous fit constrains the solution and introduces redundancy, enhancing resistance to noise. By leveraging information across the entire speckle contrast versus exposure time curve, MESI can average out noise and avoid the spurious jumps that plague single-exposure readings.

### 3.2 Accuracy of Relative Blood Flow Estimation: Single-Exposure LUT Versus MESI

To quantify how accurately each method captures blood flow changes, we calculated the percentage change in estimated flow relative to a baseline BFI of $1.0\times 10^{-8}\;{\mathrm{cm}}^{2}/s$. We considered three representative flow change scenarios: a 50% decrease, a 50% increase, and a 100% increase relative to this baseline. These changes were evaluated using both the multi-exposure approach (MESI) and a single-exposure LUT approach at the three single exposure times (0.1, 1, and 5 ms). In addition, we quantified the effect of a β mismatch to the accuracy of estimated flow changes because the LUT approach requires β as an input for the data inversion.

**Fig. 5:**
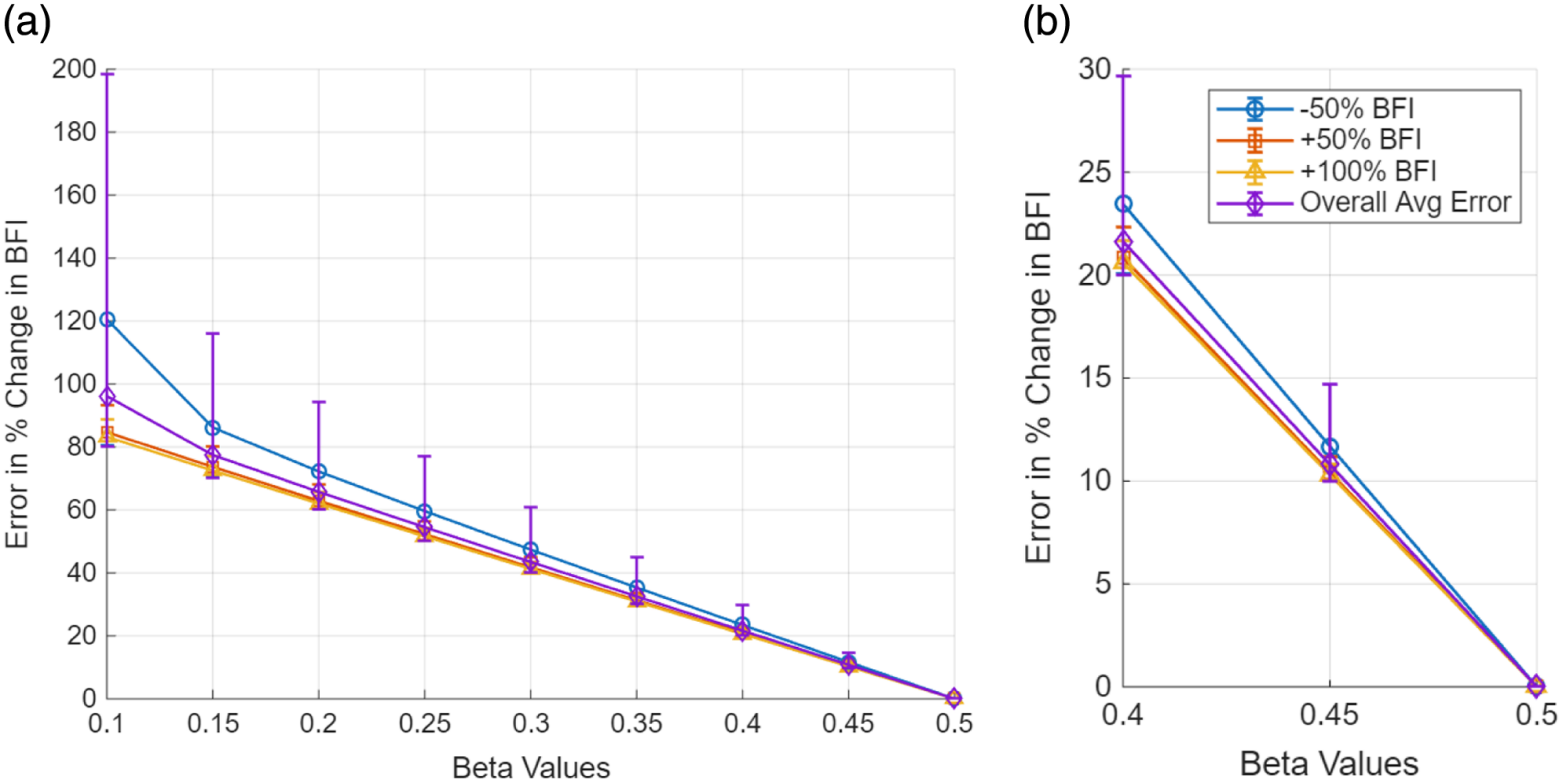
(a) Error in % change in BFI estimated with LUT, for different β mismatches, for three levels of BFI changes: −50%, +50%, and +100% relative to baseline. The X-axis indicates level of β mismatch, i.e., actual β in simulated data, β=0.5 is used for the LUT inversion. Error is calculated as the absolute deviation of the recovered % BFI change from the true change. The “Overall Avg Error” (purple line) is the mean error across all three BFI change scenarios at each β mismatch. These results correspond to the no-noise condition and are averages over the three single exposure durations. (b) Focuses on the β=0.40 to 0.50 interval, which reflect realistic β changes during an experiment. This figure demonstrates that a ±0.05 to 0.10 variation in β (consistent with realistic experimental variation) produces ∼10 to 15% error in estimated flow.

Figure 5 shows the error (zero noise condition) in estimated % change in blood flow for different levels of β mismatch with the LUT-based single-exposure method. Here, the error is calculated as the absolute deviation of the recovered % BFI change from the expected change, for each exposure time, and were averaged. As described earlier, β=0.5 was used in the LUT inversion, on simulated data (no noise) the β varied from 0.5 down to 0.1. The three flow change scenarios are plotted: −50% BFI decrease (blue ○), +50% BFI increase (orange □), and +100% BFI increase (yellow △). Error is essentially zero when the assumed β equals the true β (rightmost points at β=0.5), as expected. However, as the actual β deviates to lower values (for instance, due to motion artifacts, laser instabilities, or coupling mismatches), the single-exposure LUT method increasingly misestimates the BFI changes. A true −50% BFI increase (yellow △) is severely underestimated if the actual β is much lower than assumed with errors exceeding 120% when β=0.1. However, a +100% BFI change (blue ○) is somewhat less sensitive to β error, showing roughly 90% error at β=0.1. The overall average error across all three scenarios illustrates that even a moderate β mismatch (e.g., true β=0.4) leads to a significant ∼20% mean error in the estimated flow change. These results highlight a critical limitation of single-exposure methods that rely on a fixed β: even small deviations from the assumed β value can amplify errors in estimated blood flow changes.

**Fig. 6:**
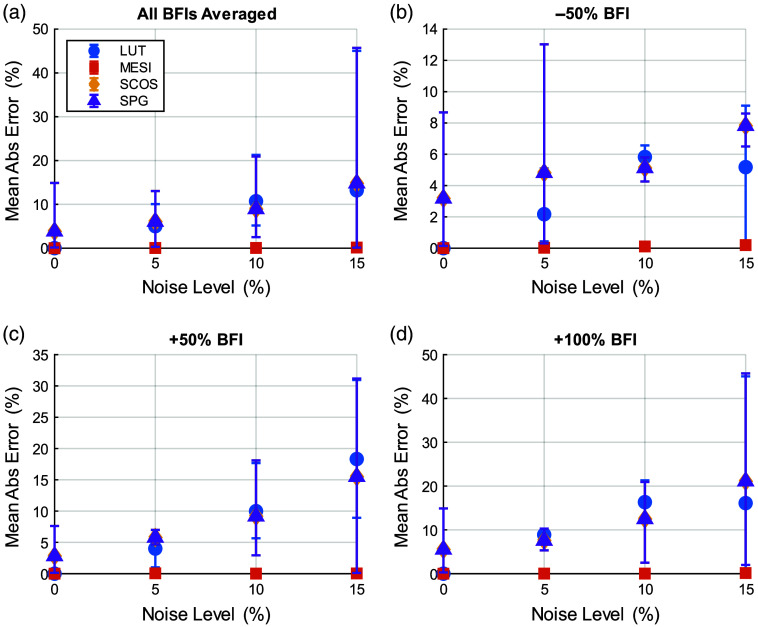
Mean absolute error in recovered % BFI changes as a function of noise for four estimation methods—LUT (circle), MESI (square), SCOS (diamond), and SPG (triangle)—with error bars denoting the full range of simulation trials and markers indicating the trial mean. (a) Error averaged across all non-baseline BFI changes (−50%, +50%, and +100%). (b) −50% change. (c) +50% change. (d) +100% change.

We also compared how all four estimators (MESI, LUT, SCOS, and SPG) fared in measuring changes in blood flow. Figure 6 summarizes how each method’s mean absolute error grows with added speckle‐noise, but with no β bias. Even at 0% noise, LUT and MESI recover flow changes with near-zero bias, whereas the single‐point inversions (SCOS and SPG) both exhibit a small, identical error—because both are simple $\frac{1}{V}$-based estimates that reduce to the same calculation. As noise increases, MESI shows close to zero-error, LUT error climbs modestly, and SCOS/SPG errors track together, rising from ∼5% at 5% noise to ∼15% at 15% noise.

### 3.3 Pulsatile Flow Tracking

Finally, we evaluated how these errors translate to a physiologically relevant blood flow signal, using pulsatile BFI values adapted from a prior DCS measurement in the adult human arm. SCOS, SPG, LUT, and MESI methods were used to estimate relative blood flow time courses; the average blood flow index through the duration of the signal was considered the baseline. LUT inversion was performed on data with β=0.45 and β=0.5, simulating a small β mismatch. For SCOS and SPG, the β mismatches did not significantly impact the results because the data are normalized to a baseline. Under 0% noise, all methods align tightly with the ground truth. Introducing ±1% noise raises MESI’s max error to ≈2% and SCOS/SPG/LUT errors to 4 to 6% at blood flow peaks. At ±5% noise, MESI’s max error is ≈12%, whereas single‐exposure methods exceed ≈22% and distort waveform amplitude (flattened or exaggerated peaks).

**Fig. 7:**
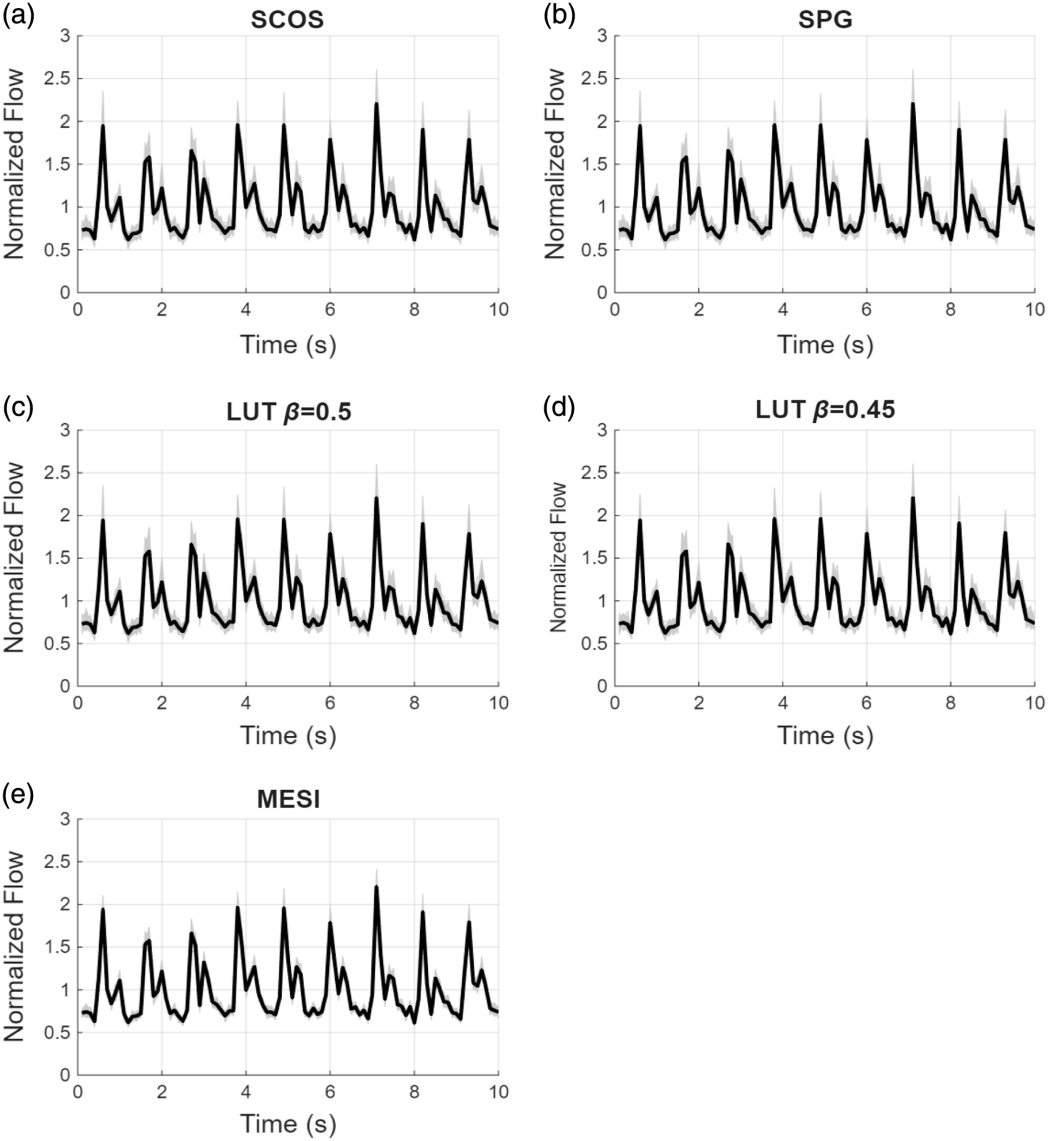
Normalized pulsatile flow waveforms estimated from 5000 trials under ±5% noise for (a) SCOS, (b) SPG, (c) LUT with no β mismatch, (d) LUT with small β mismatch, and (e) multi-exposure fits. Individual trial waveforms are represented in gray, with the mean waveform highlighted in bold black. Single-exposure results are averaged over all three exposure times. MESI shows tighter consistency and closer alignment to the true waveform shape compared with single-exposure methods.

**Fig. 8:**
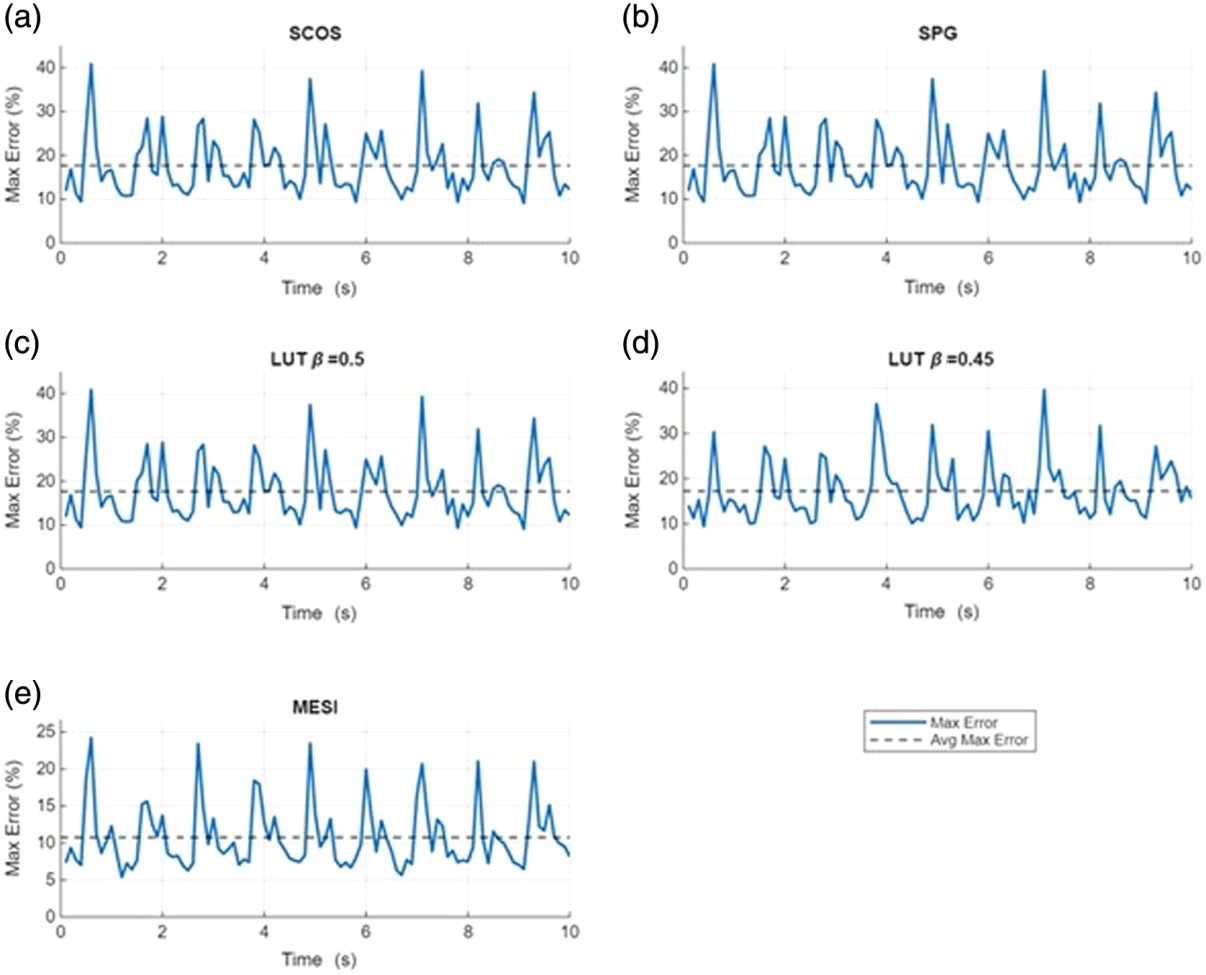
Time-resolved maximum tracking error (%) for pulsatile relative BFI recovery averaged over all three exposure times under ±5% noise conditions for (a) SCOS, (b) SPG, (c) LUT with no β mismatch, (d) LUT with small β mismatch, and (e) multi-exposure fits. Solid lines represent the instantaneous maximum errors over 5000 trials, whereas dashed lines indicate the average maximum error. MESI demonstrates consistently lower maximum error and reduced variability compared with single-exposure methods (SCOS, SPG, LUT β=0.5, and LUT β=0.45), highlighting its robustness and waveform tracking capability.

**Fig. 9:**
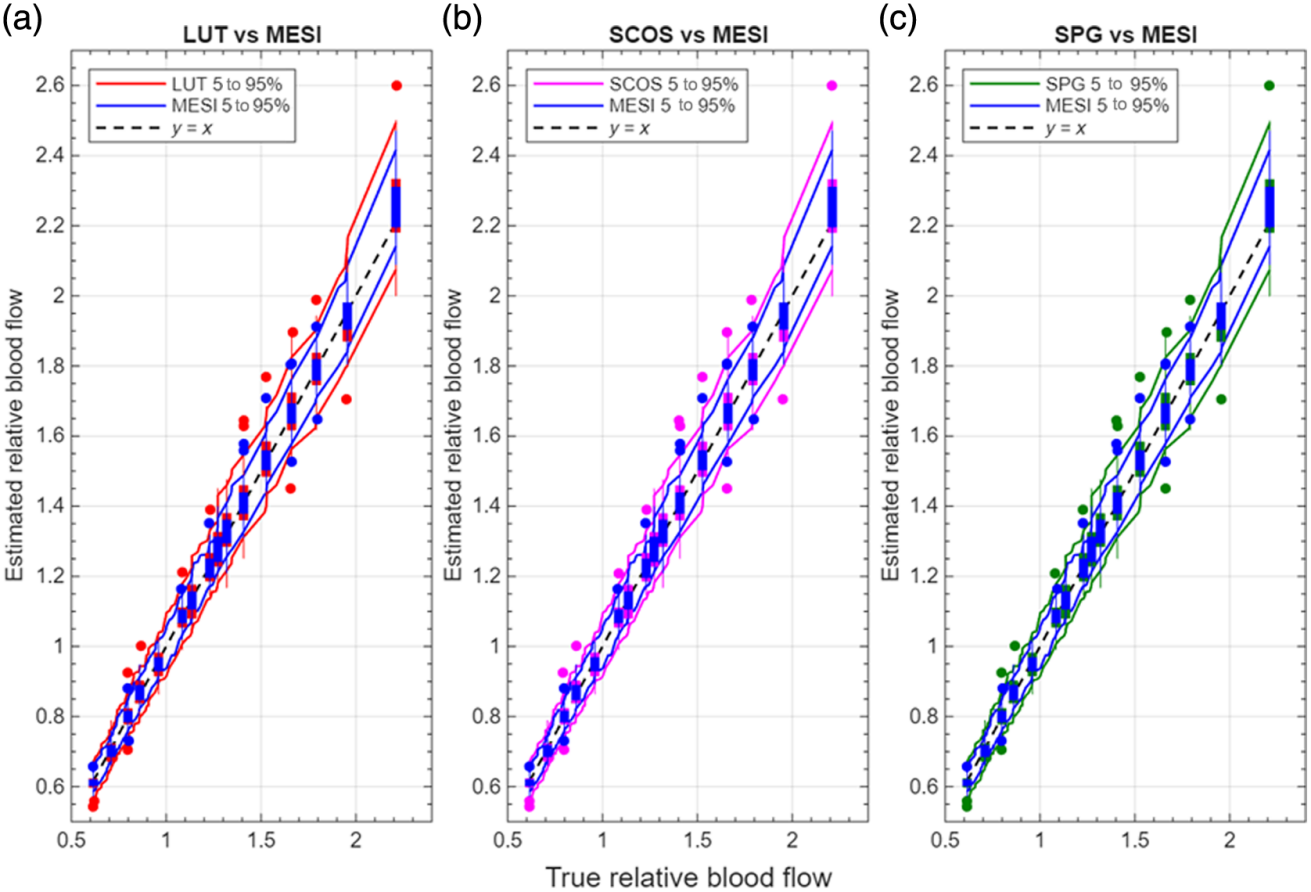
(a)–(c) Distributional comparison of normalized relative blood-flow estimates. Each panel compares a single-exposure method (red: LUT; magenta: SCOS; and green: SPG) with MESI (blue). Solid curves show the 5th to 95th percentile envelopes over 50 noisy trials; box plots at selected true-flow values summarize trial variability. The dashed line is the 1:1 line denoting perfect reproduction of relative blood flow. MESI retains the narrowest bands and best agreement with the true relative flow.

Figures 7–9 show the results of the pulsatile‐flow tracking with 5% noise. Figure 7 shows the estimated normalized blood flow waveforms for the five estimation methods; the gray-shaded region indicates the spread in blood flow estimates across the 5000 trial runs, whereas the black line is the average. Figure 8 plots the maximum % error in blood flow estimates relative to the true normalized blood flow index over the 5000 runs. For the single-exposure methods, the errors shown represent the average across all three exposure durations (0.1, 1, and 5 ms). Individual-exposure analysis showed slightly higher noise sensitivity at 0.1 ms, but similar overall trends at 1 and 5 ms; no single-exposure dominated the aggregate error. The error is highest at the blood flow peaks. Single-exposure methods may produce max errors of close to 40%; almost twice as much as MESI. The average maximum % error is ∼10.5% for MESI and ∼17.5% for the single exposure methods.

Figure 9 compares normalized estimates of relative blood flow across the three single-exposure methods (LUT, SCOS, and SPG) against MESI, plotted over 50 repeated noisy (5%) trials. A perfect estimation of the blood flow changes would map along the dashed y=x line. Deviations from this 1:1 line represent distortions in the waveform. For each method, the 5th to 95th percentile bounds and representative box plots at selected points are displayed. Box plots are shown sparsely for clarity. To emphasize differences in pulsatility shape and noise sensitivity, we report both percentile band width and trial-to-trial variance; MESI exhibits narrower envelopes and smaller variance, indicating quantitatively tighter waveform tracking despite similar mean shapes. The comparisons emphasize differences in pulsatility shape and noise sensitivity rather than global scaling errors. In all three subplots, the relative blood flow estimated with MESI is closer to the y=x line compared with the LUT, SCOS, and SPG estimations. Further, the 5th to 95th percentile band is narrower for MESI compared with the single exposure estimators. Although small trial-to-trial deviations are observed for both approaches, the MESI distributions in Fig. 9 exhibit a narrower 5th to 95th percentile band (±3% versus ±6%) and reduced variance, confirming quantitatively tighter pulsatility tracking. Finally, the deviations are greater (for all estimators) for larger flow changes (2×), which highlights that the errors in estimates of relative blood flow are most noticeable at the peaks of the pulsatile waveform. These results highlight that although normalization suppresses β mismatch bias, MESI retains the tightest error distribution, whereas single-exposure methods show increased dispersion and reduced robustness.

**Fig. 10:**
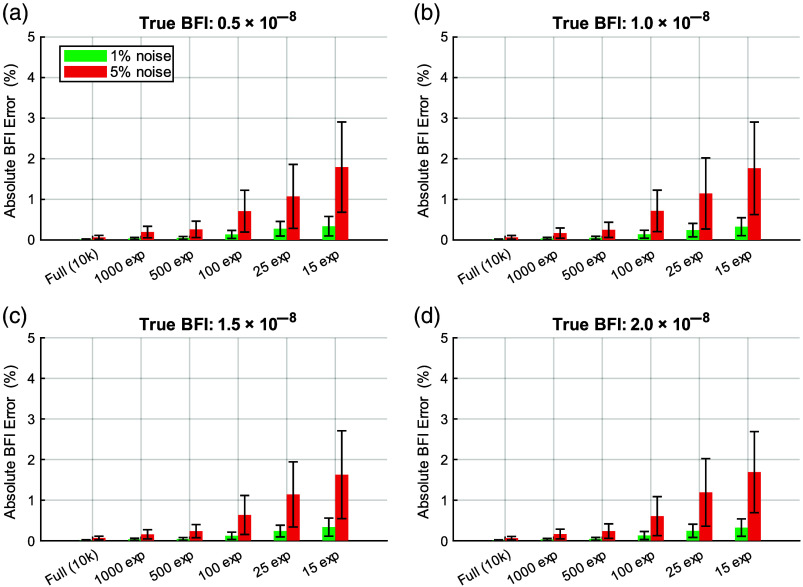
(a)–(d) Effect of reducing the number of exposures on MESI BFI estimation accuracy. Absolute BFI error (%) for four simulated flow rates under 1% (green) and 5% (red) additive noise. MESI fitting was performed using 10,000, 1000, 500, 100, 25, and 15 exposure durations. Error bars indicate the standard deviation across noise realizations. Although fewer exposures lead to slightly larger errors, the overall changes remain small, and MESI maintains accurate BFI estimation even with exposure counts typical of *in vivo* acquisition.

To assess how the number of exposure sampling influences MESI flow estimation, we evaluated BFI accuracy using exposure sets ranging from densely sampled curves (10,000 exposures) to more experimentally feasible subsets (1000, 500, 100, 25, and 15 exposures). Figure 10 shows the resulting absolute BFI error across four flow conditions under both 1% and 5% noise. As the number of exposure durations decreases, the error increases gradually, but the overall magnitude of these changes remains small. Even with only 25 or 15 exposure durations, the absolute error stays below 0.5% for 1% noise and below 3% for 5% noise across all flows tested. These results indicate that accurate estimation of the speckle-visibility curve does not require densely sampled exposure sets, and that MESI maintains stable BFI performance under exposure counts typically achievable for *in vivo* imaging. These results align with earlier studies showing that reliable MESI estimates can be obtained from a relatively small set of well-distributed exposure durations.^28^

## 4 Discussion and Conclusion

In this study, we quantified the fidelity of single exposure speckle contrast analysis to estimate baseline and relative changes in deep-tissue blood flow and compared them against a multi-exposure approach. We simulated diffuse speckle visibility curves under physiologically realistic flow conditions to test the robustness of the single- and multi-exposure approaches for different experimental and noise conditions. Our results demonstrate that MESI consistently outperforms the single-exposure methods in both accuracy and resilience to mismatch of speckle averaging factor (β) and measurement noise, maintaining <1% error where others exceed 10% to 90%. This highlights MESI’s superior accuracy and robustness for quantitative blood flow monitoring under practical conditions.

We note a few important observations from this study. We based this study on data generated from an analytical diffusion forward model with added noise. The analytical model that we used is typical for diffuse speckle blood flow measurements with a homogenous semi-infinite layer. Although this data are not experimentally measured or generated from light-transport simulations, our results from analytical models underscore a mathematical limit to how accurate single exposure methods can be in the presence of inaccurate/varying experimental parameters or noise conditions.

Incorrect or inaccurate assumptions of speckle averaging factor (β) significantly degrade the signal-to-noise ratio and measurement accuracy, arguably limiting the utility of single-exposure methods such as SCOS-, SPG-, and LUT-based approaches.^29^ Although single-exposure methods can partially mitigate β mismatch through *in situ* calibration or dual-exposure strategies, these approaches remain susceptible to inaccuracies when conditions deviate from assumptions. Our results suggest that even within a narrower β band (say 0.4 to 0.5), a miscalibration of 0.05 could introduce ∼10% error—which could be on the order of physiological changes of interest. Such small changes in β can easily occur through the course of an experiment. For example, motion of the probe can affect probe–tissue coupling and alter the distance between the skin and the detecting element, which in turn can change β. By contrast, MESI accounts/corrects for these β variations by acquiring speckle data at multiple exposure durations and fitting these to a diffusion model, thus providing more robust and reliable quantification of blood flow indices across varying experimental conditions.

Our results demonstrate that incorporating multiple exposure times (the MESI approach) offers substantial benefits in both accuracy and precision. MESI achieved accurate BFI estimates (within ∼1% of true values) across a wide range of flow changes and β values, whereas single-exposure methods showed significant biases unless β was known perfectly. MESI’s estimates remained stable with increasing noise. By contrast, single-exposure methods (whether using a fixed analytic equation or a LUT) suffered from significant systematic errors if β was not exactly known, and their performance degraded more with noise. Further, we show that no single exposure time is optimally sensitive to all flow speeds. MESI, by fitting a multi-point visibility curve, provides quantitative flow measures. For example, in these simulations, MESI correctly reflected a −50%, +50%, and +100% flow change with <1% error, whereas a 1-ms single exposure yielded only approximately −23%, +25%, and +50% changes for the same conditions. This improvement is in line with prior reports that multi-exposure speckle imaging extends the range over which flow measurements are linear and accurate.^25^

Pulsatile flow tracking simulations further highlight MESI’s advantages over single-exposure methods. Under increasing noise levels, MESI maintains tighter waveform fidelity and lower average maximum errors (<12% at ±5% noise), whereas single-exposure approaches exceed 22% error and exhibit waveform distortions, such as flattened or exaggerated peaks. We note that error for relative blood flow estimated with MESI is higher for the pulsatile flow when compared with the steady state estimates with MESI. One possible reason could be that the pulsatile blood flow represents a range of relative blood flows rather than a single steady state value, and thus, the error in relative blood flow estimates may deviate more than in steady state. Further, the results in Figs. 7 and 9 show that the mean error in relative blood flow estimated with MESI is low (about on par with the steady state conditions). Figure 8 shows the maximum error in relative blood flow estimates across the 5000 noisy simulations, i.e., some isolated trials in the noisy data could have contributed to the high max errors. Figure 9 further illustrates these differences in pulsatility shape and noise sensitivity through the 5th to 95th percentile envelopes and variance metrics: MESI demonstrates narrower distributions and reduced trial-to-trial variability compared with the single-exposure methods. Although minor deviations are observed for all methods, their frequency and magnitude are significantly lower for MESI, consistent with its tighter error distribution. The narrow error distribution of MESI across trials contrasts with the broad dispersion of single-exposure estimates, especially during rapid systolic peaks. These distortions could cause changes in the morphological features of the blood flow waveform which could be important for disease diagnoses,^30^ especially when combined with machine learning techniques. These results demonstrate MESI’s robustness for dynamic flow monitoring, reinforcing its clinical potential in applications requiring precise tracking of pulsatile blood flow changes.

Despite the robust findings presented, several limitations of this study should be acknowledged. First, although simulations captured realistic noise levels and physiologically relevant optical parameters, actual biological tissue variability might introduce complexities not fully captured here, such as spatial heterogeneity, non-uniform blood flow, and dynamic physiological fluctuations. Future studies should extend MESI validation through extensive experimental evaluations *in vivo*, incorporating diverse tissue types, anatomical sites, and real-time clinical conditions.

The results establish MESI as a robust approach for quantitative blood flow imaging, offering significant advantages in accuracy, range, and robustness over SCOS, SPG, and single-exposure LUT-corrected approaches. MESI’s ability to produce reliable, calibrated flows with minimal error will be especially beneficial in contexts such as cerebral blood flow monitoring in stroke or trauma,^26^^,^^31^ where accurate perfusion quantification is essential.

## Data Availability

All data in support of the findings of this paper are available within the article. The code will be made available upon request.
